# TBK1 Facilitates GLUT1-Dependent Glucose Consumption by suppressing mTORC1 Signaling in Colorectal Cancer Progression

**DOI:** 10.7150/ijbs.70742

**Published:** 2022-05-09

**Authors:** Diyuan Zhou, Yizhou Yao, Liang Zong, Guoqiang Zhou, Min Feng, Junjie Chen, Ganggang Liu, Guoliang Chen, Kang Sun, Huihui Yao, Yu Liu, Xinyu Shi, Weigang Zhang, Bo Shi, Qingliang Tai, Guanting Wu, Liang Sun, Wenqing Hu, Xinguo Zhu, Songbing He

**Affiliations:** 1 Department of General Surgery, the First Affiliated Hospital of Soochow University, Suzhou, Jiangsu 215006, China.; 2 Department of Gastrointestinal Surgery, Changzhi People's Hospital, The Affiliated Hospital of Changzhi Medical College, Changzhi, Shanxi 046000, China.; 3 Department of Gastrointestinal Surgery, Changshu No. 2 Hospital, Suzhou, Jiangsu 215006, China.; 4 Department of Biochemistry and Molecular Biology, Soochow University Medical College, Suzhou, Jiangsu 215123, China.; 5 Department of General Surgery, the Affiliated Hospital of Jiangsu University, Zhenjiang, Jiangsu 212000, China.; 6 Department of Medicine, Soochow University Medical College, Suzhou, Jiangsu 215006, China.

**Keywords:** TBK1, GLUT1, Colorectal cancer, mTORC1, Autophagy

## Abstract

Intestinal inflammation is a vital precipitating factor of colorectal cancer (CRC), but the underlying mechanisms are still elusive. TANK-binding kinase 1 (TBK1) is a core enzyme downstream of several inflammatory signals. Recent studies brought the impacts of TBK1 in malignant disease to the forefront, we found aberrant TBK1 expression in CRC is correlated with CRC progression. TBK1 inhibition impaired CRC cell proliferation, migration, drug resistance and tumor growth. Bioinformatic analysis and experiments *in vitro* showed overexpressed TBK1 inhibited mTORC1 signaling activation in CRC along with elevated GLUT1 expression without inducing GLUT1 translation. TBK1 mediated mTORC1 inhibition induces intracellular autophagy, which in turn decreasing GLUT1 degradation. As a rescue, blocking of autophagosome and retromer respectively via autophagy-related gene 7 (ATG7) or TBC1 Domain Family Member 5 (TBC1D5) silence diminished the regulation of TBK1 to GLUT1. GLUT1 staining presented that TBK1 facilitated GLUT1 membrane translocation which subsequently enhanced glucose consumption. Inhibitor of TBK1 also decreased GLUT1 expression which potentiated drug-sensitivity of CRC cell. Collectively, TBK1 facilitates glucose consumption for supporting CRC progression via initiating mTORC1 inhibition induced autophagy which decreases GLUT1 degradation and increases GLUT1 membrane location. The adaptive signaling cascade between TBK1 and GLUT1 proposes a new strategy for CRC therapy.

## Introduction

Colorectal cancer (CRC) is currently the third most commonly diagnosed cancer and accounts for 9% of all cancer-related deaths with the continuing rise of incidence in young people. The various pathogenic factors of CRC have been elaborated by numerous studies 1, 2. Among these pathogenic factors, innate immune system disorder and associated chronic intestinal inflammation have been defined as major factors 3-5. The innate immune system plays a key role in the continuous defense of the intestines from external stimuli and pathogen invasions. Immune system maladjustment could induce an overactivated inflammatory reaction leading to chronic intestinal inflammation, which has been thought to trigger CRC. However, the underlying mechanisms need further elucidation 3.

TANK-binding kinase 1 (TBK1) is a member of the non-canonical IkappaB kinase (IKK)-related innate immune kinase family that is well-defined as a mediator of the expression of interferon-stimulated genes (ISGs). Located downstream of various pattern recognition receptors (PRRs), TBK1 initiates the transcription of inflammatory factors against incursive pathogens. TBK1 can also respond to an intrinsic leakage of DNA/RNA, which indicates genetic damage that related to mutation and oncogenesis. In addition to promoting inflammatory gene transcription, TBK1 plays vital roles in other signaling pathways, including the protein kinase B (AKT/PKB) survival signaling, mechanistic target of rapamycin kinase (mTOR), and epidermal growth factor receptor (EGFR) pathways, which are also closely regulated by TBK1 to support cancer development 6-14.

The interaction of TBK1 with mTORC1 achieved our attention, as one of the two distinct complexes of mTOR, mTORC1 has direct effect on the enhancement of tumorigenesis by regulating cell growth in response to nutrients and growth factor signaling, through its substrates including ribosomal protein S6 kinase 1 (S6K1) and the translational repressor eIF4E-binding protein (4EBP1) 15-20. mTORC1 signaling tightly regulates glucose metabolism in cancer cells by promoting aerobic glycolysis characterized with elevated glucose uptake and high rates of glycolysis in the presence of oxygen, supporting cancer cell growth and proliferation. The increased uptake of glucose is usually accompanied by a high expression of glucose transporter 1 (GLUT1), which is the main rate-limiting factor in the transport of glucose in cancer cells. Numerous studies have reported high GLUT1 expression in human solid tumors, which promotes oncogenesis 21. In view of the multiple functions of TBK1 in immune signaling, metabolic transformation, and oncogenesis, we predicted that activated TBK1 caused by chronic intestinal inflammation may promote the development of CRC by facilitating metabolic transformation 22. A more thorough understanding of the metabolic signaling pathways in cancer cells and immune signaling that promotes metabolism transitions may contribute to improvements in cancer therapy 7.

Previous study demonstrated TBK1 was involved in cancer development by regulating metabolism transformation, but the mechanism was still undefined. Our present study revealed the aberrant TBK1 expressed in CRC tissues, TBK1 closely regulates mTORC1 signaling activation followed with the change of autophagy which mediates GLUT1 function promoting CRC development. TBK1 could be a therapeutic target in CRC by method that interrupt glucose metabolism.

## Materials and methods

### Patients and Tissue Specimens

CRC tissues and adjacent paracancer tissues were collected between 2017 and 2019 from patients diagnosed with CRC at the First Affiliated Hospital of Soochow University (Suzhou, China) without artificial selection. Forty pairs of samples were used for immunohistochemistry (IHC) and western blotting (WB) analysis. All patients met the following criteria: (a) Postoperative pathological diagnosis of colorectal adenocarcinoma with no other history of malignant tumors; (b) No history of chemotherapy, radiotherapy, or other oncotherapy before surgery; (c) Complete clinical data were available and patients agreed to be followed up; (d) All patients were informed of this study and provided written informed consent. Samples were stored in liquid nitrogen or formalin-fixed. The clinical stages and pathological features of CRC were defined according to the criteria of the American Joint Commission on Cancer. All experiments involving human subjects were performed in accord with the relevant guidelines and regulations of Soochow University and the Code of Ethics of the World Medical Association (Declaration of Helsinki). The study was approved by the Biomedical Research Ethics Committee of the First Affiliated Hospital of Soochow University.

### Immunohistochemistry (IHC) Staining

The paraffin-embedded tissues were cut into 5-μm sections and then deparaffinated with xylene and ethyl alcohol (100%, 95%, 80%, 70%) and washed with distilled water and phosphate-buffered saline (PBS). The sections were then stained with hematoxylin for 3 min, followed by a wash with tap water. A 0.5% hydrochloric acid ethanol solution was added for several seconds, and the sections were then washed again with tap water. Staining with eosin solution for 1 min and a tap-water wash were then conducted. Dehydration with ethyl alcohol (70%, 80%, 95%, 100%) and xylene followed. The tissues were finally sealed with neutral resins.

The expression of TBK1 in the tumor tissue and paracancer normal tissue was evaluated by IHC as described 23, 24. The immunoreactive scores (IRSs) were the product of the percentage of positive cells (0: <5%, 1: 5%-25%, 2: 25%-50%, 3: 50%-75%, 4: >75%) multiplied by the staining intensity (0: negative, 1: weak, 2: moderate, 3: strong). The IRS was then classified as negative (0-1), weakly positive (2-3), moderately positive (4-7), or strongly positive (8-12). We recorded the IRSs of 0-4 as negative and 5-12 as positive. The IHC results were evaluated by two pathologists separately.

### Cell Culture and Treatment

Human HT-29, HCT116, SW480, SW620 and LOVO CRC cell lines were obtained from the Cell Bank of the Chinese Academy of Sciences (Shanghai, China). HT-29 and LOVO cells were cultured in Roswell Park Memorial Institute (RPMI) 1640 medium (Gibco, Grand Island, NY, USA), and the HCT116, SW480 and SW620 cells were cultured in Dulbecco's modified Eagle medium (DMEM, Hyclone, Logan, UT) supplemented with 10% fetal bovine serum (FBS) (Biological Industries, Beit Haemel, Israel) and 1% antibiotics penicillin/streptomycin (Invitrogen, Waltham, MA). The cells were cultured at 37 °C in a 5% CO_2_ humidified atmosphere. Rapamycin (RAPA, Apexbio, Houston, TX), 5-fluorouracil (5-FU, Sigma Aldrich, St. Louis, MO) and amlexanox (Abcam, Cambridge, England) were dissolved in dimethyl sulfoxide (DMSO, Sigma Aldrich). Poly (I:C) (Apexbio) was dissolved in H_2_O.

### Plasmids

PcDNA3.1-Myc-vector, pcDNA3.1-Myc-TBK1 plasmids encoding human wild-type (WT) TBK1 and TBK1-specific short hairpin RNA (shRNA) plasmid were obtained from the Public Protein/Plasmid Library (Nanjing, China). PcDNA3.1-GFP-GLUT1 and PcDNA3.1-HA-GLUT1 were gifts from Professor Xiong Su (Soochow University Medical College). The plasmid sequences were verified via Sanger sequencing. Plasmids were transfected into cells using Lipofectamine™ 2000 Transfection Reagent (Thermo Fisher Scientific, Waltham, MA) according to the manufacturer's instructions. TBK1-specific short hairpin RNA (shRNA) plasmid was packaged with psPAX2 lentivirus-packaged vector and PMD2G lentivirus envelope plasmid (gift from Professor Xiong Su) in HEK293T cells by using polyethylenimine (Sigma-Aldrich, Missouri, USA) according to the manufacturer's instruction.

### Transfection of small interfering RNA/short hairpin RNA (shRNA)

FAM NC siRNA, NC siRNA and three different pairs of TBK1-specific siRNA oligonucleotides were designed and purchased from IBSBIO (Shanghai, China). HCT116 and SW480 cells were transfected with siRNA with Lipofectamine™ RNAiMAX Transfection Reagent (Invitrogen) at a final concentration of 50 nM according to the manufacturer's instructions. The sequences specific for human TBK1 were selected based on their potency to inhibit the target gene expression (Suppl. Table S1). Lentivirus particles were transfected into CRC cells in the presence of 6 μg/ml polybrene. Stable cell lines were further selected with 0.5 μg/ml puromycin (Sigma-Aldrich, Missouri, USA) for 2 weeks.

### Antibodies and Reagents

TBK1/NAK (D1B4) rabbit monoclonal (#3504), mTOR (#2972), phospho-mTOR (Ser2448) (#2971), 4E-BP1 (#9452), phosoho-4E-BP1(Thr37/46) (#2855), LC3A/B (#12741) and phospho-p70 S6 Kinase (Thr389) (D5U1O) rabbit monoclonal antibodies (#97596) for immunoblotting were obtained from Cell Signaling Technology (CSTS, Danvers, MA). P70 S6 kinase rabbit monoclonal antibody (#E175) was obtained from Abcam. TBK1 rabbit antibody (#DF7026) used for IF/IHC was obtained from Affinity Biosciences (Cincinnati, OH). GAPDH mouse monoclonal (#AF0006), α-tubulin rabbit polyclonal (#AF0001), β-actin mouse monoclonal (#AF0003) and horseradish peroxidase (HRP)-labeled goat anti-mouse antibodies (#A0216) were procured from Beyotime Biotechnology (Shanghai, China). The P62 rabbit antibody, TBC1D5 rabbit antibody, ATG7 rabbit antibody and HRP-labeled goat anti-rabbit IgG(H+L) (#A0208) were obtained from ProteinTech (Wuhan, China).

### Protein Extraction and Western Blotting Analysis

Cells were washed twice with ice-cold PBS and harvested with RIPA lysis buffer (Sigma Aldrich) for 30 min at 4 °C. Whole protein extraction was performed according to the manufacturer's protocol, and the cell lysates were stored at -80 °C for further experiments. The WB process was as described 23, 24.

### RNA Extraction and Quantitative Real-Time PCR (qRT-PCR)

Total RNA was extracted from cells using TRIzol Reagent (Invitrogen, Life Technologies, Carlsbad, CA) according to the manufacturer's protocol. First, 1 μg RNA was reverse transcribed using a RevertAid First Strand cDNA Synthesis Kit (Thermo Fisher Scientific). The qRT-PCR was performed using Power SYBR^®^ Green PCR Master Mix (Applied Biosystems, Foster City, CA) on the 7500 real-time PCR system (Applied Biosystems) according to the manufacturer's instructions. Fold changes were calculated relative to 18-S (internal control) using the 2^-ΔΔ^CT method. The primers used are listed in Supplementary Table S2.

### Cell Viability Assay

Cell viability was determined using CCK-8 (#1018, Apexbio) according to the recommendations of the manufacturer. Cells were seeded in 96-well plates. After the supernatant of each well was removed and the cells were washed twice with warmed PBS, 10 μL of CCK-8 solution mixed with 100 μL complete medium was added to each well. After a further 1-4 h of incubation, the absorbance was measured at 490 nm using a microplate reader (Bio-Rad, Hercules, CA). Each treatment was analyzed in triplicate.

### Cell Migration and Scratch assay

Cell invasion and migration assays were performed using Transwell plates (8 mm, 24-well format; Corning Inc., Corning, NY). Cells (2×10^4^) were suspended in 200 μL of serum-free medium and placed in the upper compartment of each chamber, followed by the addition of 700 μL of culture medium with 10% FBS to the lower chamber as a chemoattractant. Cells that invaded the lower chamber were stained with 0.5% crystal violet after 24 h of incubation. The chamber was cultured at 37 °C in a 5% CO_2_ incubator for 24 h. Cells were imaged using a microscope (200×), and the level of migration was quantified by counting the number of invaded cells in five random regions per specimen by ImageJ software ver. 1.4.3.

For the scratch wound assay, cells were seeded into six-well plates and cultured with a complete medium until confluent. Cells were scratched with a plastic tip across the center of the chamber in a straight line, and then five images were randomly acquired at 0 h and 24 h after scratching; the distances of migrated cells were then measured under a light microscope. The scratch areas were quantified by ImageJ software ver. 1.4.3.

### Bioinformatic Analysis

The dataset for CRC samples with corresponding survival data was obtained from the Cancer Genome Atlas (TCGA) database. The expression analysis of TBK1 and GLUT1 in normal and tumor tissues was performed with a Gene Expression Profiling Interactive Analysis (GEPIA) (http://gepia2.cancer-pku.cn/#index). A Gene Set Enrichment Analysis was performed using the GSEA software (https://www.broadinstitute.org/gsea/) according to the instructions given on that website. Gene The get was obtained from the GEO database (GSE94543).

### Immunofluorescence (IF)

Cells were fixed in 4% paraformaldehyde at room temperature for 15 min and permeabilized with 0.5% Triton X-100 for 10 min. The cells were then rinsed with PBS-glycine, blocked with 5% goat serum in PBS, and incubated overnight with primary antibodies at 4 °C in blocking buffer. The cells were then washed, incubated with Alexa Fluor-488 (1:300, ProteinTech) for 60 min at room temperature, washed, and mounted using Prolong Gold Anti-Fade mounting medium (Thermo Fisher Scientific).

For the localization analysis of HCT116 and SW480 cells expressing GFP-GLUT1, cells were transfected with GFP-GLUT1^WT^ plasmid, fixed and permeabilized as mentioned above, and immunostained with anti-GLUT1. Confocal microscopy was performed at ambient temperature using a 40× or 60× (NA: 1.49; oil) objective on a Leica inverted microscope, solid-state 488 (for Alexa Fluor 488) and 460 lasers (for DAPI), and a CoolSNAP MYO cooled scientific-grade CCD camera. The fluorescence colocalization of TBK1 and mTOR was technically supported by Wuhan Saiweier Biotechnology Co., Ltd.

### Glucose Uptake

Cells were seeded into a 24-well plate at a density of 1×10^5^ cells/well and after the treatments, the cells were exposed to 0.1 mM 2-NBDG (2-(N-(7-Nitrobenz-2-oxa-1, 3-diazol-4-yl) Amino)-2-Deoxyglucose) (Invitrogen) in the culture medium. Plates were incubated at 37 °C with 5% CO_2_ for a period of time as described in the experiment. Images were obtained using identical acquisition settings on a fluorescence microscope (Leica). The mean fluorescence intensity was analyzed by Image J software.

### Subcutaneous Xenograft

BALB/c nude mice (SPF grade, 16-18 g, 2-3 weeks old, male) were purchased from Shanghai Silaike Laboratory Animal Co. The mice were housed in a pathogen-free room with a 12-h light/dark cycle. A total of 2×10^6^ cells were inoculated into the back of nude mice by subcutaneous injection, the tumors were harvested 3 weeks after injection. All animal experimental procedures were approved by the Animal Ethics Committee of Soochow university.

### Statistical Analyses

All experiments were performed at least three times, and the data are presented as mean ± standard error of the mean (SEM). Significant differences were evaluated using one-way ANOVA or the Mann-Whitney U test with GraphPad Prism (version 7.0, GraphPad Software) and Statistical Package for Social Science software (version 22.0, SPSS). The IHC analysis was performed using the chi-square (χ^2^) statistical test. The χ^2^ test was used to assess the patients' clinical information with the differential expression of TBK1. The Log-rank test was used for survival analysis. The correlation between two variables was assessed using Spearman's rank correlation test. Values of p less than 0.05 were considered significant.

## Results

### TBK1 is Upregulated and Correlated with Advanced TNM Stage in CRC

**Figure 1:**
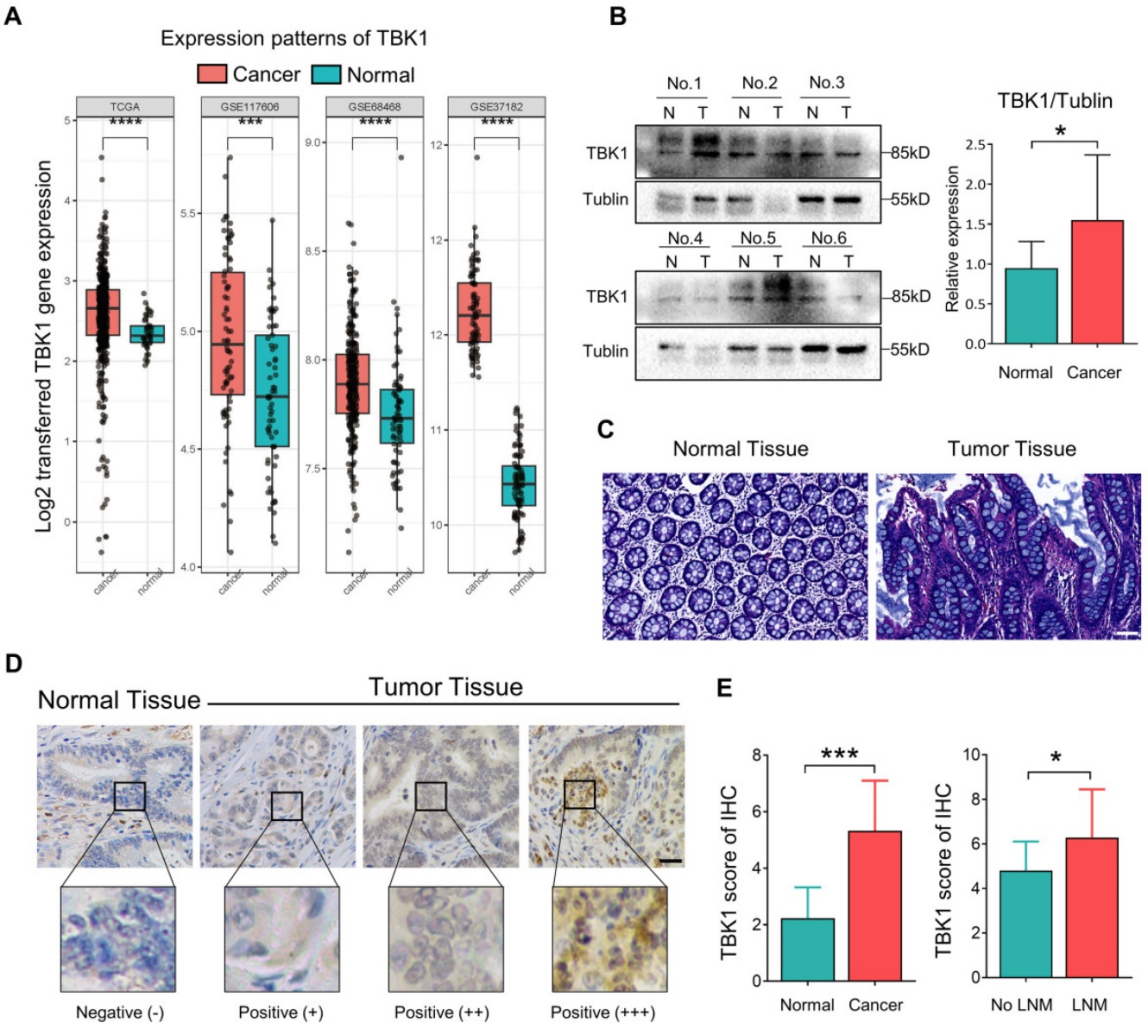
** Aberrant TBK1 expression in CRC. (A)** Differential TBK1 gene expression between CRC and normal samples from the TCGA database and three published microarray datasets (GSE117606, GSE68468, GSE37182). **(B)** WB analysis of TBK1 protein in tissue lysates from six randomly selected paired specimens. CRC tumors (T); normal tissues (N). **(C)** HE staining of normal tissue and CRC tissues. **(D)** IHC staining of TBK1 in representative normal and CRC tissues; scale bar = 50 µm. **(E)** Analysis of TBK1 IHC scores in normal tissues and CRC tissues and TBK1 IHC scores in CRC tissues with or without lymph node metastasis (LNM). *P<0.05, ***P<0.001, ****P<0.0001.

TBK1 activation by aberrant intestinal inflammation could be involved in the origin of CRC 10, 25-27. Herein, the microarray dataset-based assay between normal tissues and CRC tissues revealed a significant increase in the TBK1 mRNA level in CRC tissues compared to that in normal tissues (**Figure 1A**). We further compared the TBK1 expression by western blotting (**Figure 1B**) and IHC (**Figure 1D, E**) between colorectal tumor tissues and paracancer tissues (**Figure 1C**). The expression of TBK1 was increased in CRC tissues compared to the paracancer tissues (**Figure 1E, Table 1**), and the TBK1 expression in the CRC cases with lymph node metastasis was also significantly higher than those without lymph node metastasis (**Figure 1E**, **Table 2**). Consistent with the above-described results, the pooled analysis of CRC and normal tissues across 14 datasets also revealed a significant upregulation of TBK1 in CRC tissues (**Figure S1**).

The relationship between TBK1 expression levels and the clinicopathological characteristics of the CRC patients was evaluated, which revealed that the upregulation of TBK1 was significantly correlated with tumor size, lymph node metastasis and TNM stage (**Table 2**). However, there was no significance regarding age, gender, histological differentiation, tumor location, depth of invasion, metastasis or venous or neural invasion (**Table 2**). Collectively, these results indicated that increased TBK1 expression may contribute to the progression of CRC which is correlated with unfavorable prognosis of CRC patients.

### TBK1 Promotes Tumor Progression and Drug Resistance in CRC

**Figure 2:**
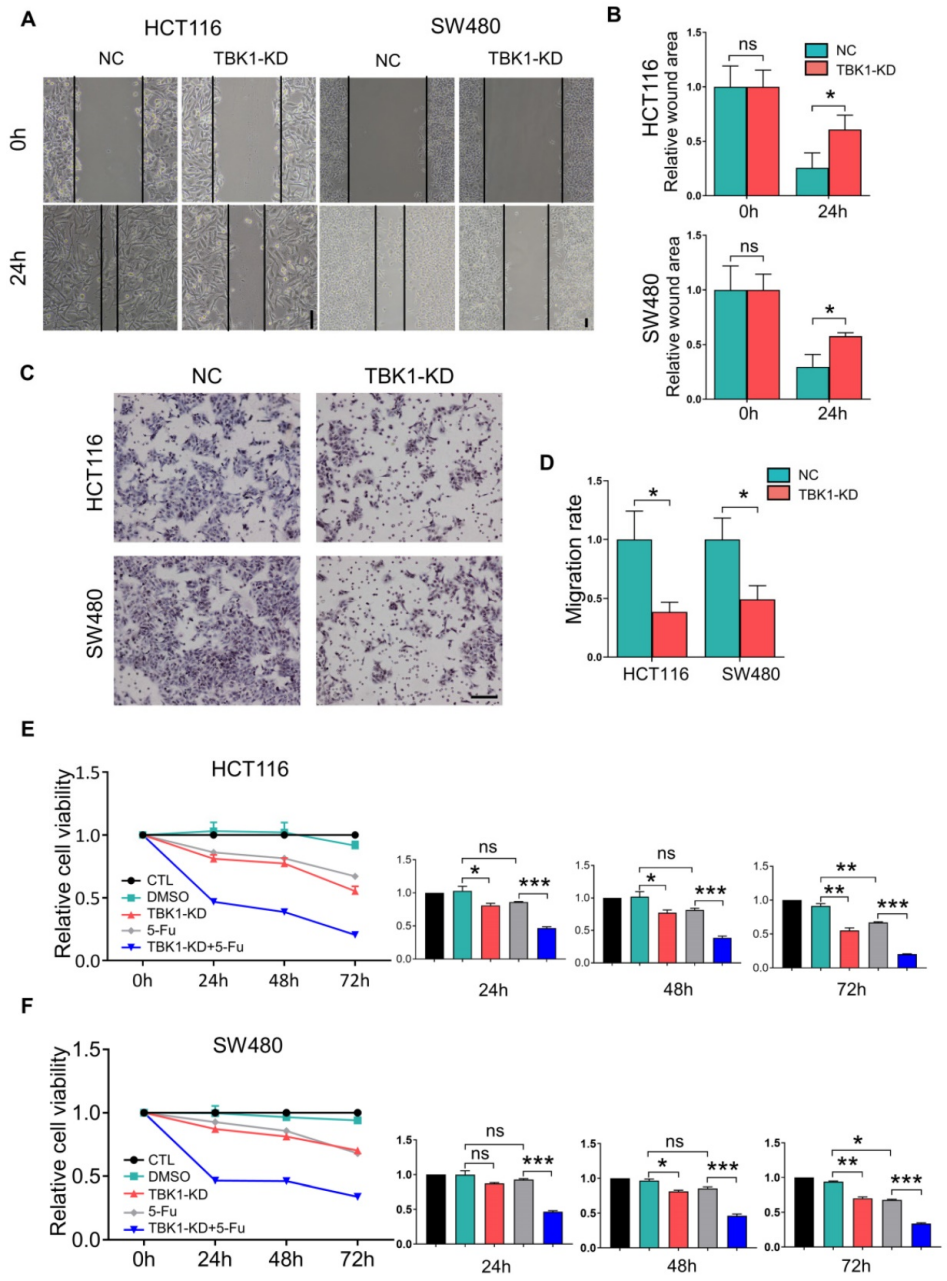
** TBK1 depletion suppressed cell migration, proliferation and drug resistance in CRC. (A)** Representative photographs of scratch wound assay of HCT116 and SW480 cells transfected with NC-siRNA or TBK1-siRNA. (scale bar = 50 µm). **(B)** The quantification analysis of the relative scratch area, mean ± SD (n=3). **(C)** Representative photographs of transwell assay of HCT116 and SW480 cells transfected with NC-siRNA or TBK1-siRNA. (scale bar = 50 µm). **(D)** The quantification of the migratory cell rate, mean ± SD (n=3). The relative cell viabilities of HCT116 **(E)** and SW480 **(F)** cells were tested with a CCK-8 assay, mean ± SD (n=3). CTL: transfected with NC-siRNA, 50 nM; DMSO: treated with DMSO; si-TBK1: transfected with TBK1-siRNA, 50 nM; 5-FU: treated with 5-FU; si-TBK1+5-FU: transfected with TBK1-siRNA+5-FU.

**Figure 3:**
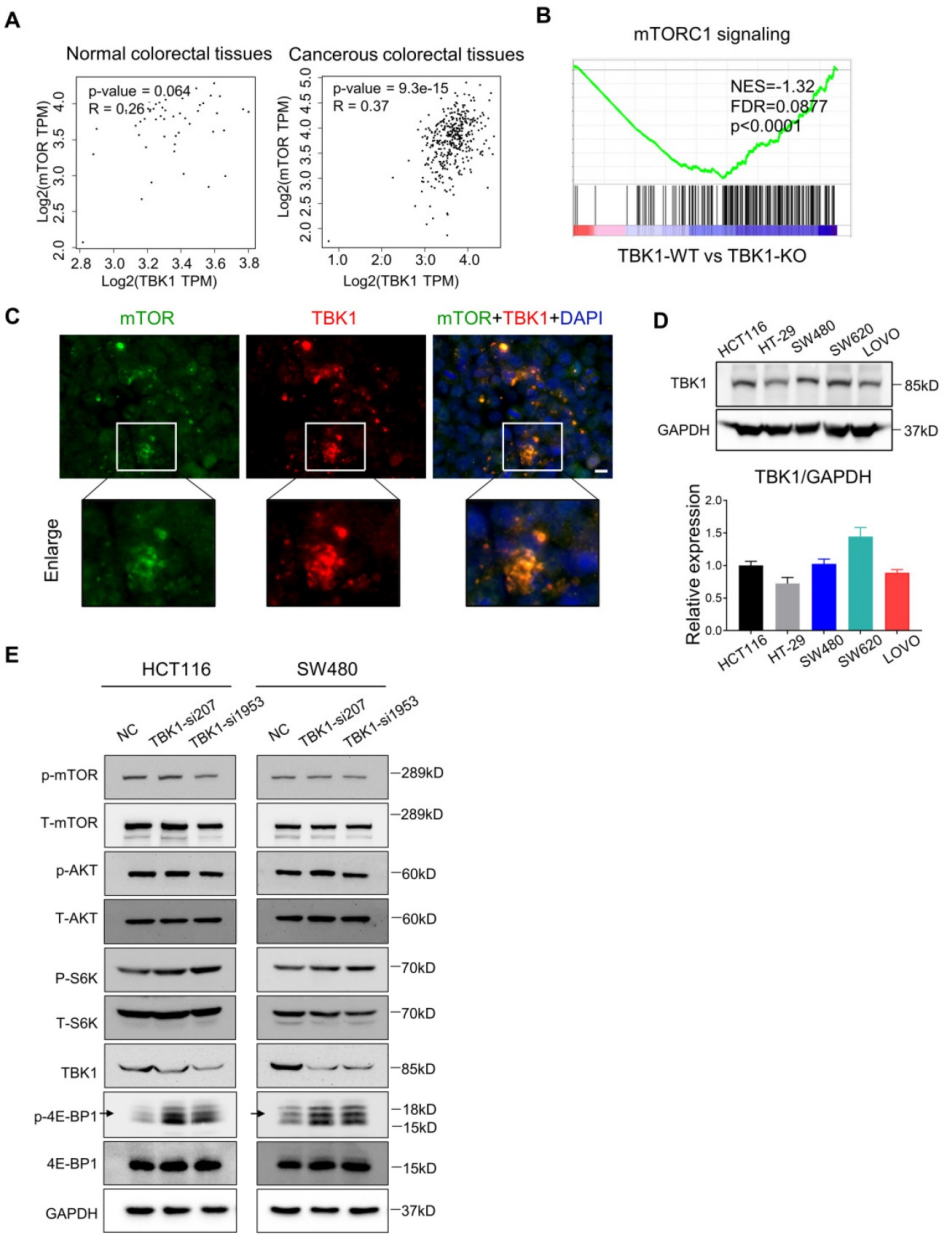
** TBK1 restrained the mTORC1 signaling activation in CRC. (A)** Correlation analysis of TBK1 and mTOR in normal tissues and CRC tissues based on the TCGA database. **(B)** GESA analyses of gene sets for mTORC1 signaling. NES: normalized enrichment score; FDR: false discovery rate. Negative NES indicates lower expression in TBK1-WT to TBK1-KO. **(C)** The expression of TBK1 and mTOR in CRC cells revealed by IF. *Green:* mTOR; *red:* TBK1; *blue:* DAPI; scale bar: 50 µm. **(D)** The expression of TBK1 in the five CRC cell lines (HCT116, HT-29, SW480, SW620, LOVO). TBK1 expression was quantified by the gray-scale value of straps. **(E)** HCT116 cells and SW480 were transfected with NC-siRNA, TBK1-si207 and TBK1-si1953, TBK1, T-mTOR, p-mTOR, AKT, p-AKT, T-S6K1, p-S6K1, 4E-BP1, p-4E-BP1 and GAPDH were analyzed by WB.

We then examined the effects of TBK1 in the development of CRC. The expression of TBK1 was evaluated in several CRC cell lines (**Figure 3D**), HCT116 and SW480 which have relatively similar and higher TBK1 expression were used for functional studies. The TBK1 expression in CRC cell lines was knocked down by TBK1-siRNA, TBK1-si207 exhibited the most effective suppression (**Figure S2**). The evaluation of cell migration and invasion ability showed that TBK1 knockdown clearly decreased cell migration (**Figure 2A, B**) and invasion (**Figure 2C, D**) abilities compared to the negative control (NC) groups. We also noted a clear reduction in cell proliferation ability after the RNA interference of TBK1 as the time passed (**Figure 2E, F**).

An earlier study reported that TBK1 could be a target for cancer adjuvant therapy 12, then we further investigated the effects TBK1 on the drug sensitivity of CRC cells by transfecting HCT116 and SW480 cells with NC-siRNA or TBK1-siRNA combined with 5-FU to explore whether TBK1 could be a potential drug target in CRC therapy. As expected, the CRC cells with TBK1 suppression were more sensitive to 5-FU (**Figure 2E, F**). Collectively, these results indicated that TBK1 could comprehensively enhance aggressive phenotypes of CRC in diverse ways including improved drug resistance.

### TBK1 Restrains mTORC1 Signaling in CRC

Recent research established mTORC1 as the core molecule downstream of TBK1 in tumorigenesis, and mTORC1 serves as a master regulator of cell metabolism, growth, proliferation and survival, which has a central role in oncogenesis 28 29 . In our present study, the express correlation analyses showed that overexpressed TBK1 was seemed to be positively correlated with mTOR specifically in CRC tissues rather than normal tissues (**Figure 3A**). The GESA results revealed an increased activation of mTORC1 signaling following TBK1 depletion (**Figure 3B**).

Hasan et al. reported a direct interaction between the TBK1 and mTORC1 complexes 6. Using IF, we observed the colocalization of TBK1 and mTOR in CRC cells (**Figure 3C**). We then manipulated the expression of TBK1 in CRC cells to assess the change in mTORC1 and mTORC2 signaling (**Figure 3E**). The downregulation of TBK1 had moderate change of phosphorylated-mTOR (Ser2448) and phosphorylated-AKT (Ser473), but remarkable increase of phosphorylated-S6K1 (Thr389) and phosphorylated-4E-BP1 (Thr36/47) were observed which are downstream of mTORC1 (**Figure 3E**). These results suggested that mTORC1 was regulated by TBK1 in a different manner in CRC.

**Figure 4:**
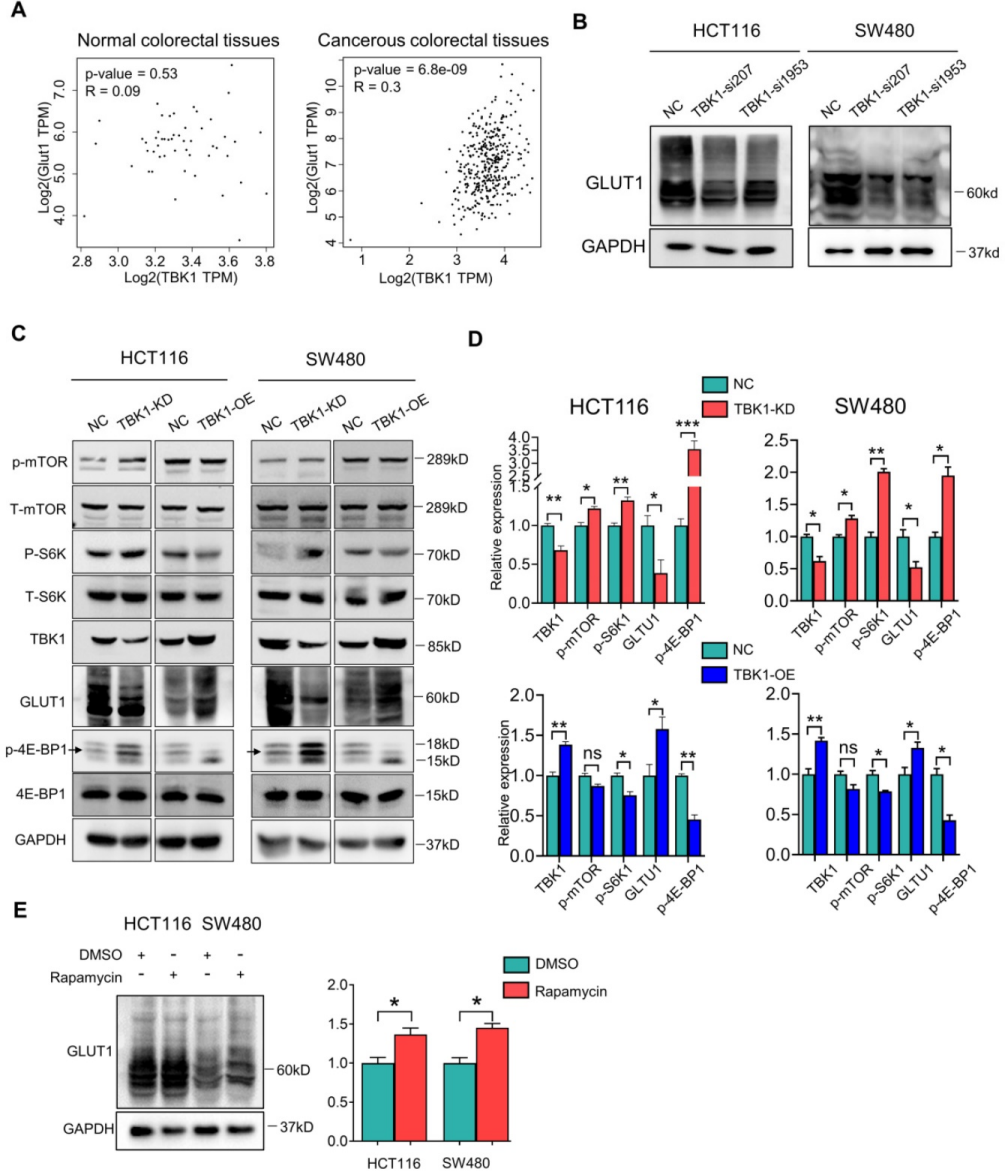
** The inhibition of mTORC1 signaling increased the GLUT1 expression in CRC. (A)** Correlation analysis of TBK1 and GLUT1 in normal colorectal and CRC tissues based on the TCGA database. **(B)** The lysates of HCT116 and SW480 transfected with two TBK1 siRNAs were blotted for GLUT1 and GAPDH. **(C)** HCT116 and SW480 cells transfected with NC-siRNA, TBK1-siRNA, vector plasmid and TBK1^WT^ plasmid as indicated, the cell lysis was immunoblotted. **(D)** The expression of TBK1, p-mTOR, p-S6K1, GLUT1and p-4E-BP1 were quantified. Data are mean ± SD (n=3) NC: negative control; TBK1-KD: TBK1 knockdown; TBK1-OE: TBK1 overexpression. **(E)** HCT116 and SW480 were treated with rapamycin (5 µM) for 12 h, the lysis was blotted and the GLUT1 expression was quantified. *P<0.05, **P<0.01.

To further determine the influence of TBK1 in the mTORC1 signaling pathway, we transfected HCT116 and SW480 cells with a vector and TBK1^WT^ plasmid. The increased expression of TBK1 actually decreased the phosphorylation level of S6K1 and 4E-BP1, whereas the change in p-mTOR was not obvious (**Figure 4C, D**). These results indicated that TBK1 seemed to suppress the function of mTORC1 and the downstream kinase S6K1 and 4EBP1, but have no obvious effect on mTOR.

### The Suppression of mTORC1 Promotes GLUT1 Expression in CRC

TBK1 activation was reported to facilitate the transformation of cellular metabolism, which resembles the Warburg effect by suppressing mTORC1 activity 6, but the underlying mechanism has been unclear. The Warburg effect is characterized by increased glucose uptake, and GLUT1 is the main rate-limiting factor of glucose transport in cancer cells. Our preliminary study revealed a relationship between mTORC1 and GLUT1 23. We thus further investigated whether TBK1 regulates GLUT1 by suppressing mTORC1 signaling in CRC in the present study. The results of the correlation analysis showed that TBK1 is relatively positive correlated with GLUT1 in CRC tissues, and not in normal tissues (**Figure 4A**). The knockdown of TBK1 with different targeted siRNAs significantly decreased the GLUT1 level in CRC cells (**Figure 4B**).

We next investigated whether the relationship between TBK1 and GLUT1 is mediated by mTORC1, and we observed that TBK1 overexpression further diminished the activation of mTORC1 signaling and enhanced the GLUT1 expression (**Figure 4C, D**). To rule out whether other molecules downstream of TBK1 contribute to the change in the expression of GLUT1, we applied an effective inhibitor of mTORC1, rapamycin, to HCT116 and SW480 cells to examine the effect of mTORC1 inhibition on GLUT1. The WB analysis results indicated that mTORC1 inhibition could directly increase the GLUT1 expression (**Figure 4E**) following the reduction of P62 and increase of LC3II/I indicating cellular autophagy activation** (Figure S3)**. These results suggested that TBK1 could increase GLUT1 expression by inhibiting mTORC1 signaling in CRC.

### The regulation of TBK1 to GLUT1 is Autophagy-Dependent

**Figure 5:**
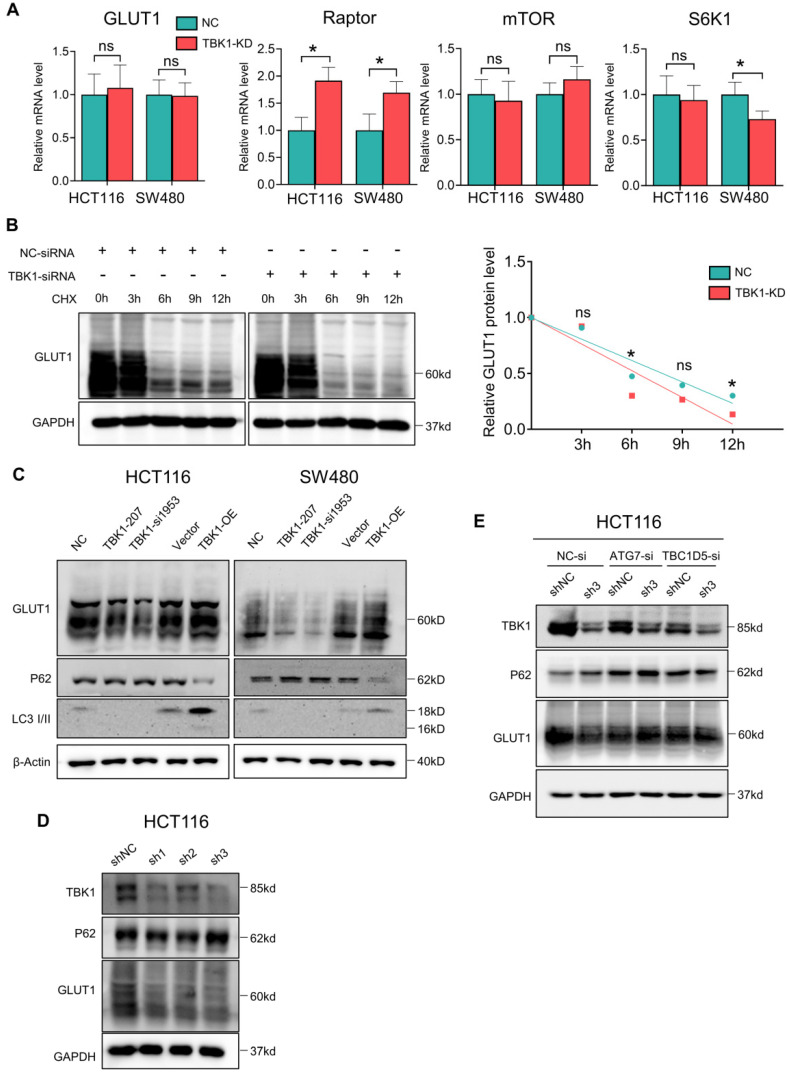
** TBK1-induced autophagy inhibited GLUT1 degradation in CRC. (A)** The mRNA levels of GLUT1, mTOR, Raptor and S6K1 in HCT116 cells transfected with NC-siRNA or TBK1-si207 for 24 h. **(B)** HCT116 cells were treated with cycloheximide (CHX) for 12 h after being transfected with NC-siRNA or TBK1-si207 for 24 h. GLUT1 and GAPDH of the cell lysates were blotted. The degradation curve is according to the relative GLIT1 grayscale value of each time point, and the bands were quantified and presented as the mean ± SD (n=3). **(C)** WB analysis of P62, GLUT1, LC3 II/I and GAPDH from whole-cell lysates. HCT116 cells were transfected with NC-siRNA, TBK1-si207, TBK1-si1953, vector plasmid and TBK1^WT^ plasmid as indicated. **(D)** WB analysis of TBK1, P62, GLUT1 and GAPDH in HCT116 cells with stable TBK1 knockdown or negative control. **(E)** Negative control (NC) and stable TBK1-knocked down HCT116 cells were separately transfected with NC-siRNA, ATG7-siRNA and TBC1D5-siRNA, and the whole cell lysates were immunoblotted for the indicated proteins.

We assessed the mRNA levels of GLUT1, mTOR, Raptor and S6K1 after TBK1 downregulation, and we observed that the Raptor transcription level was markedly increased after TBK1 downregulation, which is important for the mTORC1 complex assembly; S6K1 showed a moderate decrease. There was no significant change in the GLUT1 or mTOR transcription levels (**Figure 5A**). These results indicated that GLUT1 could be regulated post-transcriptionally, and the function of mTORC1 complex seemed to be blocked.

It is known that mTORC1 also closely regulates the intracellular process of autophagy, which is correlated with the degradation of GLUT1. Herein, the stability of GLUT1 was evaluated, and we observed that the downregulation of TBK1 resulted in an accelerated degradation of GLUT1 (**Figure 5B**). Autophagy could be the key mechanism underlying the regulation of GLUT1 by TBK1. Herein, the change of P62/SQSTM1 and LC3 II/I confirmed the inhibition of autophagy after the depletion of TBK1 in CRC cells (**Figure 5C, Figure S6A-B**), and the overexpression of TBK1 produced the opposite results (**Figure 5C, Figure S6A-B**).

We then observed that with the HCT116 cell line with TBK1 stably depleted by shRNA, TBK1-sh3 showed significant TBK1 depletion and autophagy inhibition (**Figure 5D**). Autophagy facilitates a retromer-driven translocation of GLUT1 instead of degradation in endolysosomal compartments controlled by the protein TBC1 domain family member 5 (TBC1D5) 30. Herein, the regulation of GLUT1 by TBK1 was reversed by the depletion of ATG7, which blocked the formation of the autophagosome. In addition, the knockdown of TBC1D5 also diminished the change of GLUT1 mediated by TBK1 (**Figure 5E**). Collectively, these results demonstrated that the change of cellular GLUT1 regulated by TBK1 was mediated by autophagy which arouse from mTORC1 inhibition in CRC cells.

### TBK1 Promotes GLUT1 Membrane Location and Glucose Uptake

**Figure 6:**
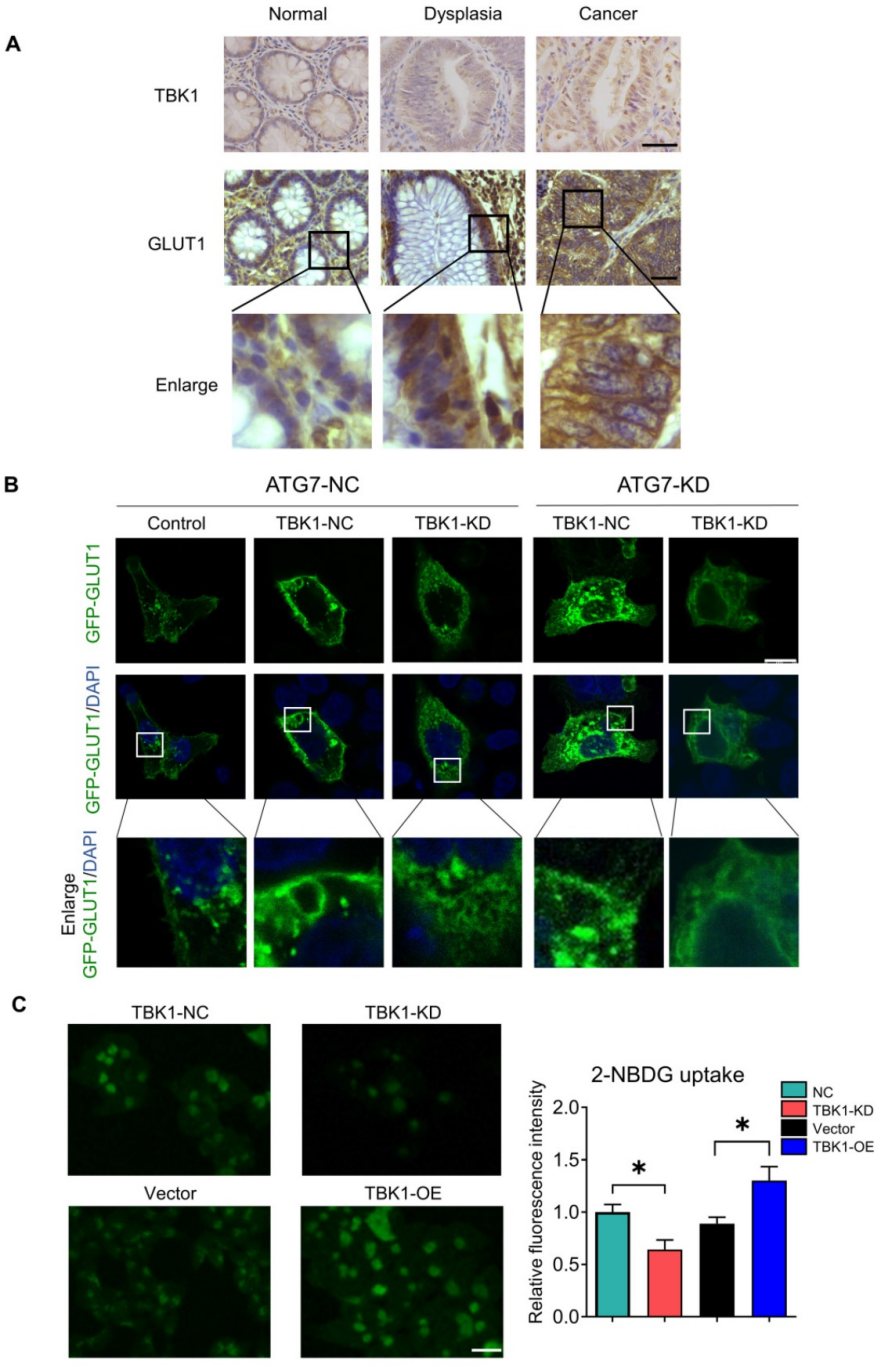
** TBK1 facilitated the cell membrane localization of GLTU1 in CRC. (A)** Representative IHC staining of TBK1 and GLUT1 in normal, dysplasia and CRC tissues (scale bar = 50 µm). *Insets:* Magnification of the boxed regions. **(B)** IF staining for GLUT1 in HCT116 cell with GFP-GLUT1 expression with indicated treatment. Scale bars: 8 µm. *Insets:* Magnification of the boxed regions. **(C)** Fluorescence images of HCT116 cells treated with 2-NBDG (100 µM) for 3h after NC-siRNA, TBK1-siRNA, Vector plasmid or TBK1^WT^ plasmid. The mean fluorescence intensities were quantified with Image J. Mean±SD, n=3, *P<0.05.

The results of the co-expression analysis revealed that high TBK1 expression was positively correlated with GLUT1 expression (**Figure 4A**). The IHC findings showed that the expression of GLUT1 increased from normal tissues to dysplasia and cancer tissues, which is consistent with the TBK1 level (**Figure 6A**). Moreover, we observed that GLUT1 had remarkable membrane localization in cancer tissues unlike dysplasia or normal tissues (**Figure 6A**). As a membrane protein, GLUT1 is located in the cell membrane, facilitating the uptake of glucose. GLUT1 has increased expression in diverse cancers exhibiting high levels of aerobic glycolysis 31, 32. The present TCGA-based analysis showed that GLUT1 was prominently expressed in CRC, and high level of GLUT1 contribute to poor disease-free survival (DFS) in CRC, but had no significant effect on overall survival (OS) (**Figure S4**).

We further evaluated the effect of TBK1 on the localization of GLUT1 in CRC cells, and the results revealed the specific localization of GLUT1 at the membrane and minor expression in the cytoplasm of CRC cells (**Figure 6B**). The downregulation of TBK1 resulted in a significant diffusion of GLUT1 in the cytoplasm instead of on the cell surface (**Figure 6B**). To further examine these results, we used ATG7-targeted siRNA to inhibit the process of autophagy, and we observed that ATG7 depletion decreased the GLUT1 localization at the membrane and restricted the regulation of GLUT1 by TBK1, which was mediated by TBK1-induced autophagy.

We next investigated whether TBK1 could alter the glucose uptake of CRC cells. The glucose uptake was significantly compromised with TBK1 downregulation compared to the negative control group and moderately enhanced by TBK1 overexpression compared to the vector group (**Figure 6C**). In brief, the above-described findings indicated that TBK1 contributes to GLUT1 membrane localization and then facilitated glucose uptake. The poor prognosis in CRC patients who have high TBK1 expression could be GLUT1-mediated 32-34.

### TBK1 is a Promising Target for CRC Treatment

**Figure 7:**
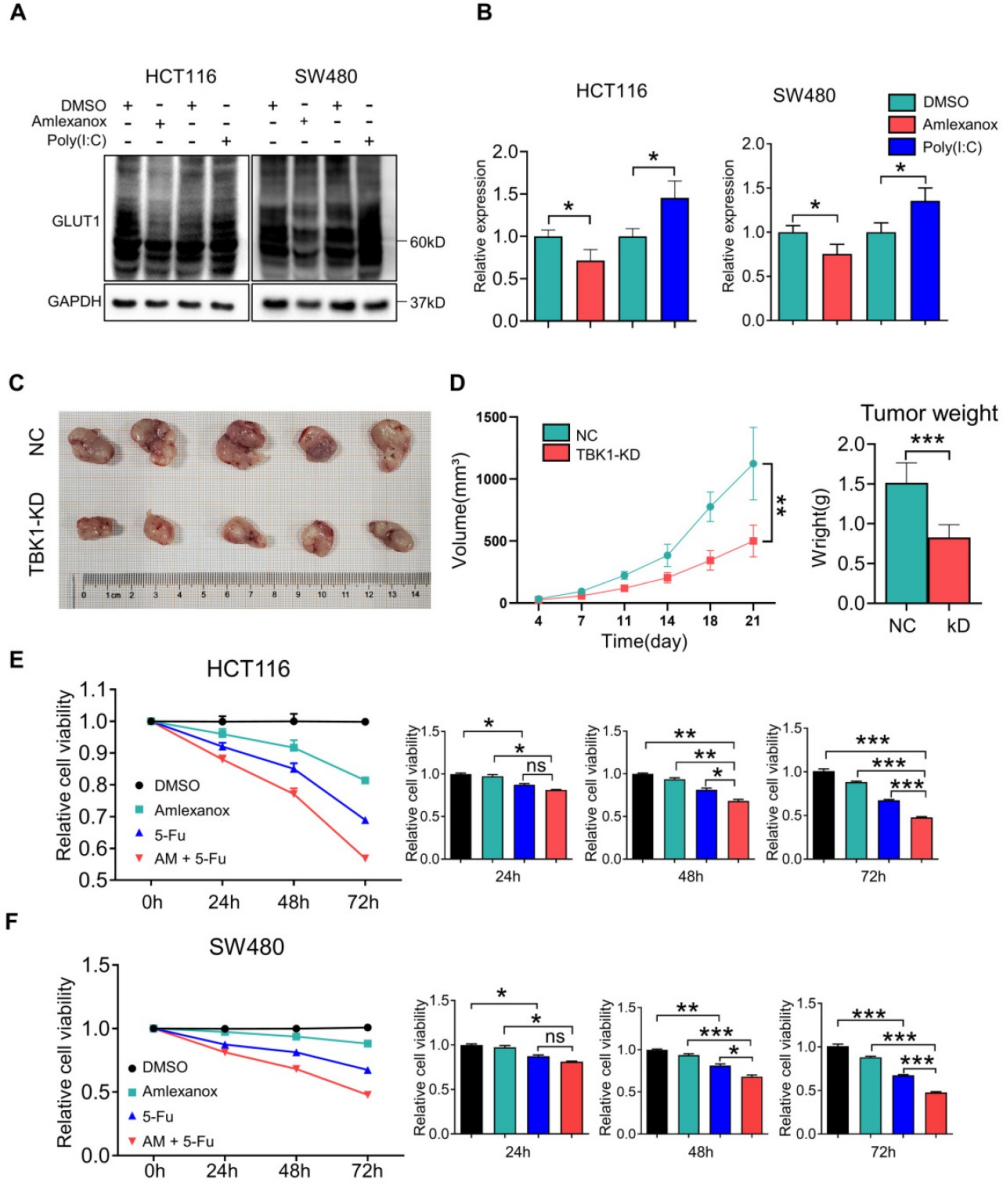
** TBK1 is a promising target for CRC treatment. (A)** HCT116 and SW480 cells were treated with amlexanox (100 µM) or poly (I:C) (2 µg/ml) for 24 h, and the lysates were blotted for GLUT1. **(B)** The GLTU1 expression was quantified (mean±SD, n=3, *P<0.05). **(C)** Representative image of tumors derived from NC-shRNA or TBK1-shRNA transfected HCT116 cells in nude mice (5/group). **(D)** Quantification of tumor volume and weight of NC and TBK1-KD groups (mean ± SD, n=5.) CCK-8 assay of HCT116 **(E)** and SW480 **(F)** cells treated as indicated. 5-FU, 5 µM; amlexanox (100 µM) for 24, 48 and 72 h. Data are mean ± SD (n=3). *P<0.05, **P<0.01, ***P<0.001.

Metabolic manipulation represents a promising therapeutic approach in cancer treatment. In view of the effects of TBK1 on GLUT1 in CRC, we next investigated the potential therapeutic function of TBK1-targeted drugs. To extend our findings, we treated CRC cells with the TBK1 inhibitor amlexanox and the immunostimulant poly (I:C) (polyinosinic: polycytidylic acid). The TBK1 inhibitor amlexanox, similar to TBK1 depletion, reduced the expression of GLUT1 and inhibited cellular autophagy with the increase of P62 and decline of LC3 II/I in HCT116 and SW480 cells, and poly (I:C) enhanced the GLUT1 expression and cellular autophagy activation (**Figure 7A-B, Figure S7**). Above results indicated that TBK1 in CRC cells maintained the response to a pharmacological inhibitor or stimulus.

We then assessed the effect of TBK1 on CRC progression *in vivo* with subcutaneous xenograft models. HCT116 cells with or without TBK1 knockdown were transplanted into nude mice (**Figure 7C**). The tumor growth from cells with TBK1 knockdown was substantially inhibited compared to that of the control group as judged by the tumor weight and tumor volume (**Figure 7D**). Amlexanox exerted a moderate inhibition of the viabilities of CRC cells (**Figure 7E-F**), but it effectively increased the curative effect of 5-FU (**Figure 7E-F**). These results indicated that the suppression of TBK1 impaired a variety of CRC cell properties and that TBK1 could be a potential therapeutic target in CRC. In conclusion, we revealed that TBK1 supports the development of CRC by augmenting a GLUT1-dependent glucose consumption through inhibiting mTORC1 induced autophagy.

## Discussion

Chronic intestinal inflammation has been regarded as playing a causal role in the origin and progression of CRC 5. The prolonged immune response gives rise to several types of immune signaling pathway, which leads to genotoxicity, hypermetabolism, immunologic derangement, aberrant proliferation and more. In the present study, we identified TBK1 as a critical kinase that facilitates the tumorigenesis of CRC by regulating glucose metabolic transformation instead of the canonical role in innate immunity.

Several studies have demonstrated that TBK1 is associated with tumorigenesis in various cancers including lung, prostate, breast, gastric cancers and melanoma 9-11, 13. Most of those studies focused on the kinase activity of TBK1 and we noted inconsistent effects of TBK1 on mTORC1 among different studies 6, 29. Aberrant expression of TBK1 in CRC tissues was observed, which is usually related to intestinal inflammation. Additionally, high TBK1 expression was correlated with large tumor, lymph node metastasis, and advanced TNM stage. The associations between aberrant TBK1 expression and aggressive clinical features in CRC patients indicated the potential effects of TBK1 in CRC development.

Previous studies have demonstrated that TBK1 has important molecular and cellular functions in addition to its classical roles in innate immune system 9, 10, 12, 13, 35. It was reported that mTORC1 interacts directly with TBK1, mTORC1 signaling is activated by TBK1 and then promotes oncogenic transformation 10, 29. In our present study, the depletion of TBK1 caused a significant decrease in the progressive phenotypes of CRC cells, and TBK1 suppressed mTORC1 signaling in CRC cells. Hasan et al. presented evidence indicating that chronic activation of TBK1 promotes cancer development by suppressing mTORC1 activity 6, 35. These results present inflammation as the key regulator of TBK1 functions, and it is possible that the mechanism whereby TBK1 mediates CRC could be modified by inflammation. Further studies are needed to illuminate how inflammation modifies the interaction of TBK1 and mTORC1.

Increasing glycolysis following chronic inflammation-induced mTORC1 inhibition is responsible for tumor development. It is known that high expression of GLUT1 is closely related to glycolysis, and our previous study also confirmed the link between mTORC1 and GLUT1 23. Herein, we examined the regulation of GLUT1 by TBK1, and we observed that TBK1 downregulation resulted in decreased GLUT1 expression, whereas the overexpression of TBK1 enhanced the GLUT1 expression. When we used rapamycin to directly inhibit mTORC1, the results confirmed that the suppression of mTORC1 signaling could increase the GLUT1 level in CRC cells, and the regulation of GLUT1 by TBK1 was mediated through mTORC1 inhibition. However, there was no significant change in GLUT1 transcription after TBK1 downregulation, indicating that GLUT1 was post-transcriptionally regulated. The chase assay of GLUT1 degradation confirmed that GLUT1 degradation was inhibited. This means that the changes in the GLUT1 level could be autophagy mediated.

In fact, mTORC1 acts as a key regulator of intracellular autophagy, we observed that TBK1 closely affects the expression of P62 and LC3II/I indicating TBK1 regulated autophagy in CRC cells. With an interruption of the autophagy process, the regulation of GLUT1 by TBK1 was reversed. The same effect was achieved by the depletion of TBC1D5, which has been reported as a mediator of GLUT1 degradation 30. Thus, the regulation of GLUT1 by TBK1 was autophagy dependent induced by mTORC1 inhibition.

Increased GLUT1 has been related to poor prognosis and neoplastic progression, and the role of GLUT1 in cancer development depends on increased glucose uptake 33, 34, 36, 37. Our present analyses demonstrated specific GLUT1 expression in cell membrane of CRC tissues, but not in that of dysplasia or normal tissues. It was reported that autophagy facilitated the cell surface localization of GLUT1 30, but no study had determined whether TBK1 could influence the localization of GLUT1. We observed that GLUT1 was localized mainly in the plasma membrane of CRC cells and a few GLUT1-positive intracellular vesicles separated in cytoplasm. In contrast, in TBK1-knockdown cells, GLUT1 was trapped mainly in cytoplasm and intracellular vesicles. The inhibition of autophagy could decrease the membrane location of GLUT1 as indicated in previous studies and glucose uptake rate of CRC was also altered with different TBK1 expression 30, 38, 39. In short, a deficiency in TBK1 could block the plasma membrane localization and stability of GLUT1 and TBK1 facilitates CRC development mediated by GLUT1. The activation or inhibition of TBK1 with pharmacological activator (Poly I:C) or inhibitor (Amlexanox) visibly affected the expression of GLUT1 in this study, and we observed that the pharmacological inhibition of TBK1 could also induce inhibition of cell proliferation and drug resistance. The CRC cells seemed to respond directly to TBK1-targeted drugs, several studies have reported the application of Amlexanox in cancer treatment and metabolism dysfunction, indicating the potential for adjuvant CRC therapy 40-43.

In summary, there is evidence that the mechanisms underlying chronic intestinal inflammation and colorectal oncogenesis converge to a certain extent. The present study provides the first report that the regulation of GLUT1 function by TBK1 is mediated by mTORC1 signaling in CRC. The autophagy induced by mTORC1 inhibition facilitates the plasma membrane localization of GLUT1 and increases GLUT1 stability. A greater understanding of the crosstalk among inflammation, metabolism transformation and cancer processes may provide new approaches for the treatment of CRC.

## Supplementary Material

Supplementary figures and tables.

## Figures and Tables

**Table 1:** TBK1 expression in colorectal tumor tissues and paracancer tissues

	TBK1	
	Positive	Negative
Tumor tissues	25	15
Paracancer tissues	1	39
χ^2^	32.821	
*P* value	<0.0001	

**Table 2:** Relationship between TBK1 expression and clinicopathological factors in CRC patients (* *P* < 0.05)

	TBK1		
	Negative	Positive	*P* value
**Age (years)**			0.251
<60	5	13	
≥60	10	12	
**Gender**			0.673
Male	10	15	
Female	5	10	
**Size (cm)**			0.033*
≤4	10	8	
>4	5	17	
**Tumor location**			0.327
Colon	6	14	
Rectum	9	11	
**Depth of tumor invasion**			0.267
T1-2	3	2	
T3-4	12	23	
**Lymph node metastasis**			0.026*
Yes	2	12	
No	13	13	
**Degree of differentiation**			0.792
Well	10	16	
Poor	5	9	
**Metastasis**			0.414
Yes	19	6	
No	13	2	
**Venous or Neural invasion**			0.273
Negative	11	14	
Positive	4	11	
**TNM staging**			0.026*
I-II	13	13	
III-IV	2	12	

## References

[1] Hernandez-Luna MA, Lopez-Briones S, Luria-Perez R (2019). The Four Horsemen in Colon Cancer. J Oncol.

[2] Brody H (2015). Colorectal cancer. Nature.

[3] Rizzo A, Pallone F, Monteleone G, Fantini MC (2011). Intestinal inflammation and colorectal cancer: a double-edged sword?. World journal of gastroenterology.

[4] Abu-Remaileh M, Bender S, Raddatz G, Ansari I, Cohen D, Gutekunst J (2015). Chronic inflammation induces a novel epigenetic program that is conserved in intestinal adenomas and in colorectal cancer. Cancer research.

[5] Elinav E, Nowarski R, Thaiss CA, Hu B, Jin C, Flavell RA (2013). Inflammation-induced cancer: crosstalk between tumours, immune cells and microorganisms. Nat Rev Cancer.

[6] Hasan M, Gonugunta VK, Dobbs N, Ali A, Palchik G, Calvaruso MA (2017). Chronic innate immune activation of TBK1 suppresses mTORC1 activity and dysregulates cellular metabolism. Proceedings of the National Academy of Sciences of the United States of America.

[7] Andrejeva G, Rathmell JC (2017). Similarities and Distinctions of Cancer and Immune Metabolism in Inflammation and Tumors. Cell Metab.

[8] Boehm JS, Zhao JJ, Yao J, Kim SY, Firestein R, Dunn IF (2007). Integrative genomic approaches identify IKBKE as a breast cancer oncogene. Cell.

[9] Deng T, Liu JC, Chung PE, Uehling D, Aman A, Joseph B (2014). shRNA kinome screen identifies TBK1 as a therapeutic target for HER2+ breast cancer. Cancer research.

[10] Cooper JM, Ou YH, McMillan EA, Vaden RM, Zaman A, Bodemann BO (2017). TBK1 Provides Context-Selective Support of the Activated AKT/mTOR Pathway in Lung Cancer. Cancer research.

[11] Xiao Y, Zou Q, Xie X, Liu T, Li HS, Jie Z (2017). The kinase TBK1 functions in dendritic cells to regulate T cell homeostasis, autoimmunity, and antitumor immunity. J Exp Med.

[12] Li J, Huang J, Jeong JH, Park SJ, Wei R, Peng J (2014). Selective TBK1/IKKi dual inhibitors with anticancer potency. International journal of cancer.

[13] Ou YH, Torres M, Ram R, Formstecher E, Roland C, Cheng T (2011). TBK1 directly engages Akt/PKB survival signaling to support oncogenic transformation. Mol Cell.

[14] Clement JF, Meloche S, Servant MJ (2008). The IKK-related kinases: from innate immunity to oncogenesis. Cell Res.

[15] Um SH, Frigerio F, Watanabe M, Picard F, Joaquin M, Sticker M (2004). Absence of S6K1 protects against age- and diet-induced obesity while enhancing insulin sensitivity. Nature.

[16] Shah OJ, Wang Z, Hunter T (2004). Inappropriate activation of the TSC/Rheb/mTOR/S6K cassette induces IRS1/2 depletion, insulin resistance, and cell survival deficiencies. Curr Biol.

[17] Makinoshima H, Takita M, Saruwatari K, Umemura S, Obata Y, Ishii G (2015). Signaling through the Phosphatidylinositol 3-Kinase (PI3K)/Mammalian Target of Rapamycin (mTOR) Axis Is Responsible for Aerobic Glycolysis mediated by Glucose Transporter in Epidermal Growth Factor Receptor (EGFR)-mutated Lung Adenocarcinoma. J Biol Chem.

[18] Poulain L, Sujobert P, Zylbersztejn F, Barreau S, Stuani L, Lambert M (2017). High mTORC1 activity drives glycolysis addiction and sensitivity to G6PD inhibition in acute myeloid leukemia cells. Leukemia.

[19] Harachi M, Masui K, Okamura Y, Tsukui R, Mischel PS, Shibata N (2018). mTOR Complexes as a Nutrient Sensor for Driving Cancer Progression. International journal of molecular sciences.

[20] Ruvinsky I, Meyuhas O (2006). Ribosomal protein S6 phosphorylation: from protein synthesis to cell size. Trends Biochem Sci.

[21] Goncalves MD, Lu C, Tutnauer J, Hartman TE, Hwang SK, Murphy CJ (2019). High-fructose corn syrup enhances intestinal tumor growth in mice. Science.

[22] Zhao P, Wong KI, Sun X, Reilly SM, Uhm M, Liao Z (2018). TBK1 at the Crossroads of Inflammation and Energy Homeostasis in Adipose Tissue. Cell.

[23] Yao Y, Yang X, Sun L, Sun S, Huang X, Zhou D (2019). Fatty acid 2-hydroxylation inhibits tumor growth and increases sensitivity to cisplatin in gastric cancer. EBioMedicine.

[24] Yao Y, Zhou D, Shi D, Zhang H, Zhan S, Shao X (2019). GLI1 overexpression promotes gastric cancer cell proliferation and migration and induces drug resistance by combining with the AKT-mTOR pathway. Biomedicine & pharmacotherapy = Biomedecine & pharmacotherapie.

[25] Durand JK, Zhang Q, Baldwin AS (2018). Roles for the IKK-Related Kinases TBK1 and IKKepsilon in Cancer. Cells.

[26] Barbie DA, Tamayo P, Boehm JS, Kim SY, Moody SE, Dunn IF (2009). Systematic RNA interference reveals that oncogenic KRAS-driven cancers require TBK1. Nature.

[27] Eskiocak B, McMillan EA, Mendiratta S, Kollipara RK, Zhang H, Humphries CG (2017). Biomarker Accessible and Chemically Addressable Mechanistic Subtypes of BRAF Melanoma. Cancer Discov.

[28] Chen GQ, Tang CF, Shi XK, Lin CY, Fatima S, Pan XH (2015). Halofuginone inhibits colorectal cancer growth through suppression of Akt/mTORC1 signaling and glucose metabolism. Oncotarget.

[29] Bodur C, Kazyken D, Huang K, Ekim Ustunel B, Siroky KA, Tooley AS (2018). The IKK-related kinase TBK1 activates mTORC1 directly in response to growth factors and innate immune agonists. EMBO J.

[30] Roy S, Leidal AM, Ye J, Ronen SM, Debnath J (2017). Autophagy-Dependent Shuttling of TBC1D5 Controls Plasma Membrane Translocation of GLUT1 and Glucose Uptake. Mol Cell.

[31] Mueckler M, Thorens B (2013). The SLC2 (GLUT) family of membrane transporters. Mol Aspects Med.

[32] Carvalho KC, Cunha IW, Rocha RM, Ayala FR, Cajaiba MM, Begnami MD (2011). GLUT1 expression in malignant tumors and its use as an immunodiagnostic marker. Clinics (Sao Paulo).

[33] Wincewicz A, Baltaziak M, Kanczuga-Koda L, Koda M, Sulkowska U, Sulkowski S (2010). GLUT1 and Bcl-xL in relation to erythropoietin in human colorectal adenocarcinomas. Hepatogastroenterology.

[34] Haber RS, Rathan A, Weiser KR, Pritsker A, Itzkowitz SH, Bodian C (1998). GLUT1 glucose transporter expression in colorectal carcinoma: a marker for poor prognosis. Cancer.

[35] Kim JK, Jung Y, Wang J, Joseph J, Mishra A, Hill EE (2013). TBK1 regulates prostate cancer dormancy through mTOR inhibition. Neoplasia.

[36] Hauptmann S, Grunewald V, Molls D, Schmitt WD, Kobel M, Kriese K (2005). Glucose transporter GLUT1 in colorectal adenocarcinoma cell lines is inversely correlated with tumour cell proliferation. Anticancer research.

[37] Zambrano A, Molt M, Uribe E, Salas M (2019). Glut 1 in Cancer Cells and the Inhibitory Action of Resveratrol as A Potential Therapeutic Strategy. International journal of molecular sciences.

[38] Eyster CA, Higginson JD, Huebner R, Porat-Shliom N, Weigert R, Wu WW (2009). Discovery of new cargo proteins that enter cells through clathrin-independent endocytosis. Traffic.

[39] Wu N, Zheng B, Shaywitz A, Dagon Y, Tower C, Bellinger G (2013). AMPK-dependent degradation of TXNIP upon energy stress leads to enhanced glucose uptake via GLUT1. Mol Cell.

[40] Liu Y, Lu J, Zhang Z, Zhu L, Dong S, Guo G (2017). Amlexanox, a selective inhibitor of IKBKE, generates anti-tumoral effects by disrupting the Hippo pathway in human glioblastoma cell lines. Cell Death Dis.

[41] Bordonaro M, Lazarova D (2019). Amlexanox and UPF1 Modulate Wnt Signaling and Apoptosis in HCT-116 Colorectal Cancer Cells. J Cancer.

[42] Bailly C (2022). The potential value of amlexanox in the treatment of cancer: Molecular targets and therapeutic perspectives. Biochem Pharmacol.

[43] Takeda K, Yano K, Yamada K, Kihara A (2021). Amlexanox enhances the antitumor effect of anti-PD-1 antibody. Biochem Biophys Res Commun.

