# Supplementary material for: TBK1 Facilitates GLUT1-Dependent Glucose Consumption by suppressing mTORC1 Signaling in Colorectal Cancer Progression
Source: Int J Biol Sci. 2022 May 9;18(8):3374–89. doi: 10.7150/ijbs.70742 (PMC9134896; doi:10.7150/ijbs.70742)
Supplement: Supplementary file 1 — Supplementary figures and tables. [file ijbsv18p3374s1.pdf]

Supplementary Material

Supplement Figure 1 Pooled analysis of TBK1 in CRC and normal tissues across 14 datasets.

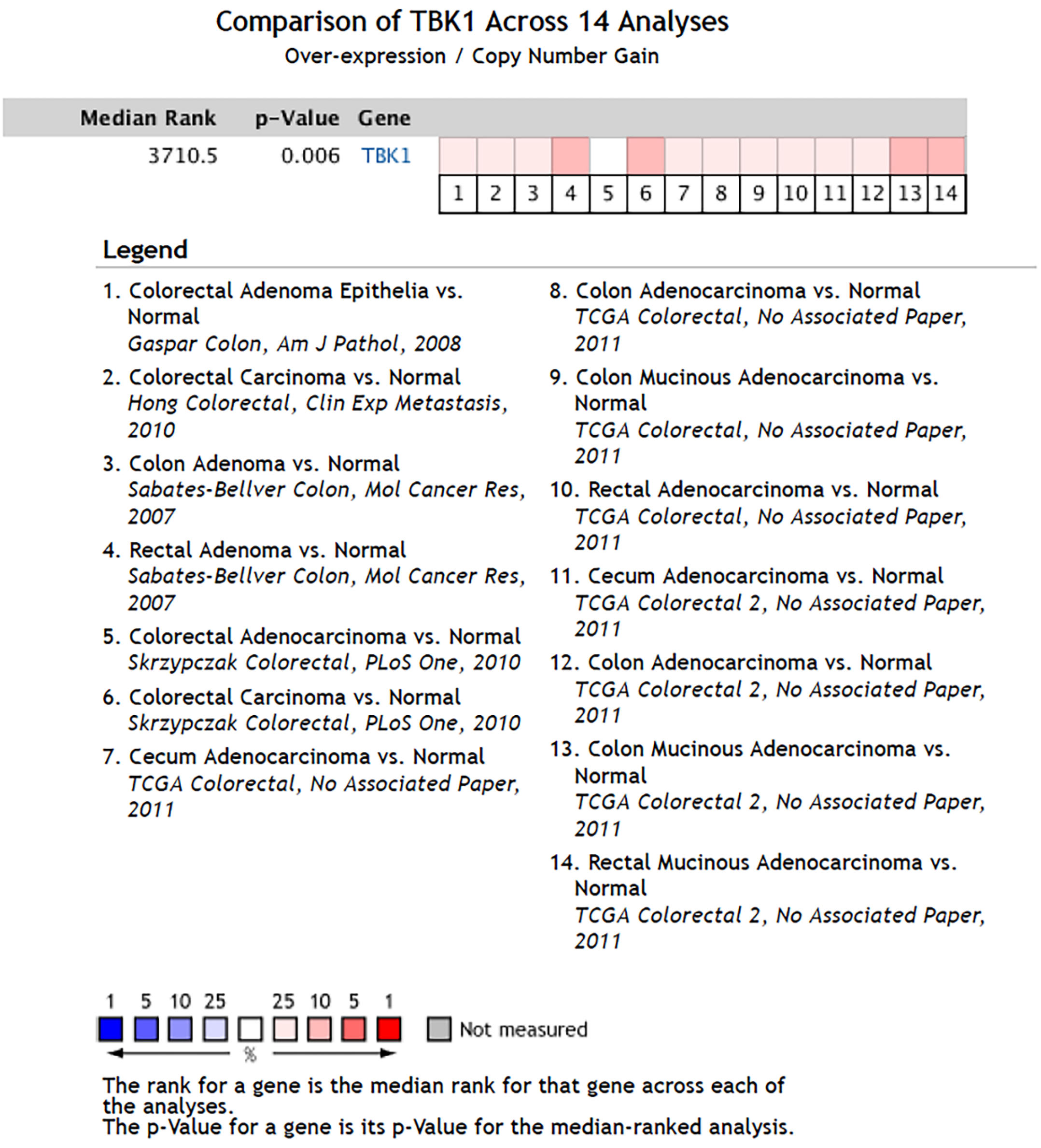

Supplement Figure 2 Efficiency analyses of TBK1-targeted siRNA (NC-siRNA, TBK1-si207, TBK1-si1310 and TBK1-si1953, 20 nM). The relative expression of TBK1 was quantified.

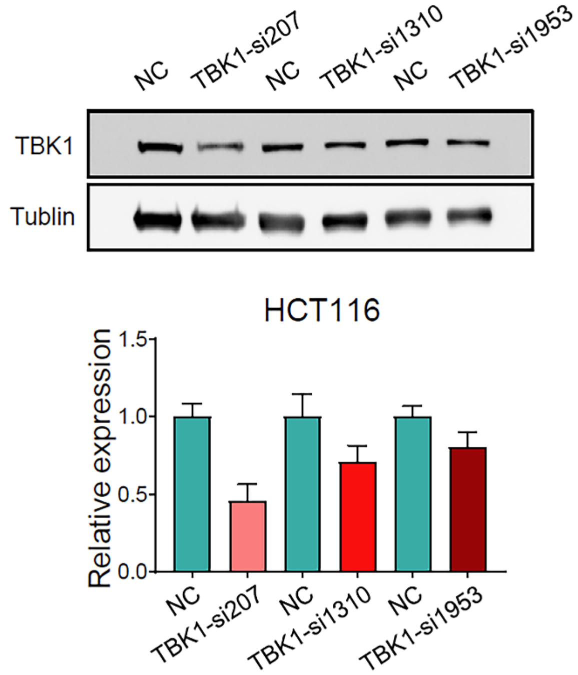

Supplement Figure 3 HCT116 and SW480 were treated with rapamycin (5 μM) for 12 h, the lysis was blotted for P62, LC3I/II and β-Actin.

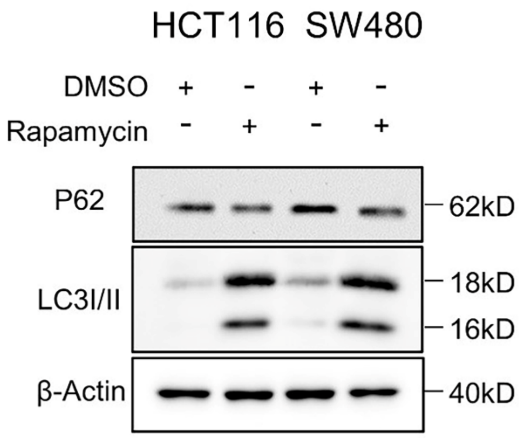

Supplement Figure 4 Expression of GLUT1 between normal and carcinoma tissues and the OS and DFS survival curves of CRC patients with high or low GLUT1 expression based on the TCGA database are shown. **P*<0.05, ***P*<0.01, ****P*<0.001.

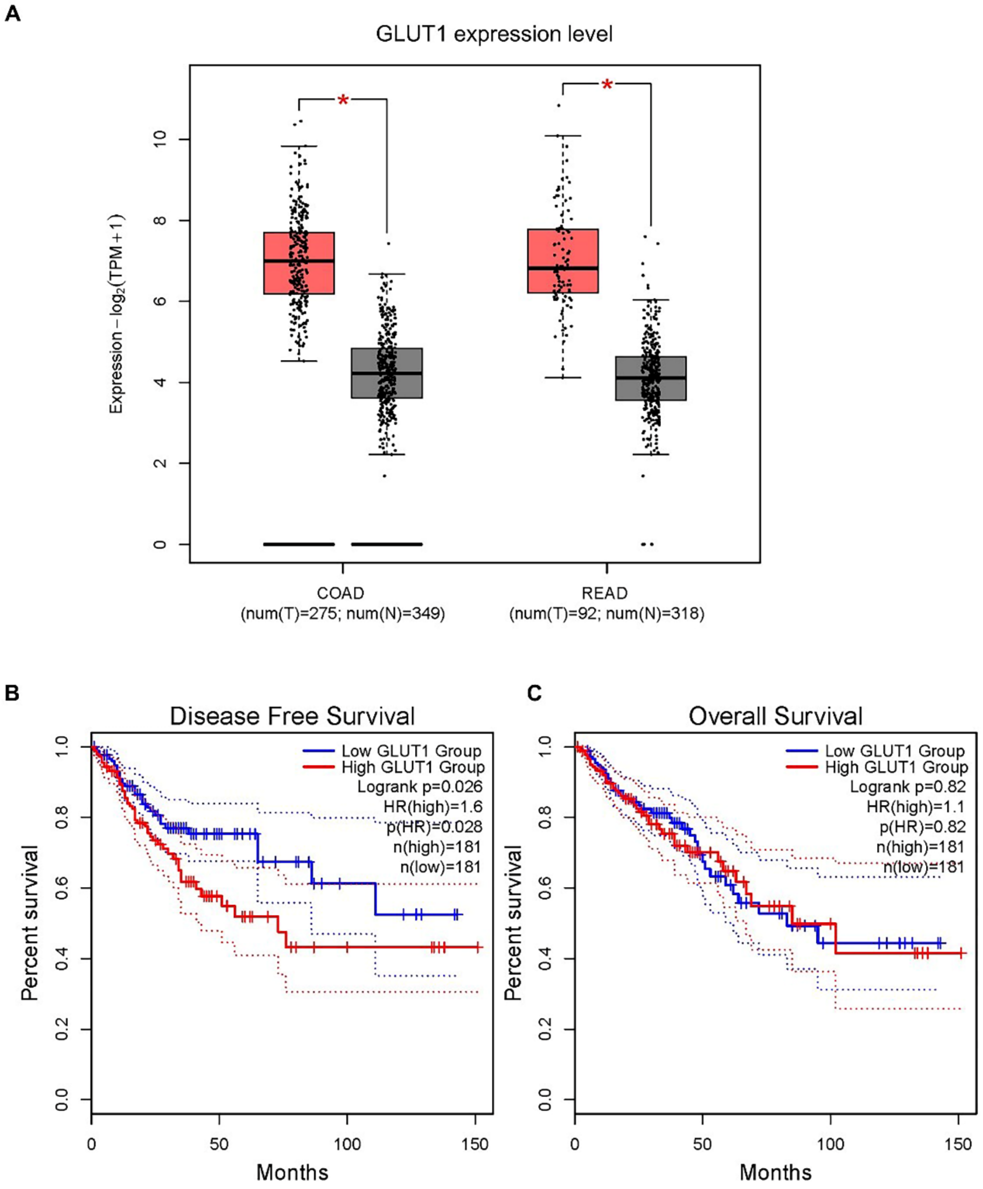

Supplement Figure 5 HCT116 were transfected with NC-siRNA, ATG-siRNA1, ATG-siRNA2, ATG-siRNA3 and TBC1D5-siRNA, the lysis were blotted for ATG7, TBC1D5, P62, LC3I/II and β-Actin.

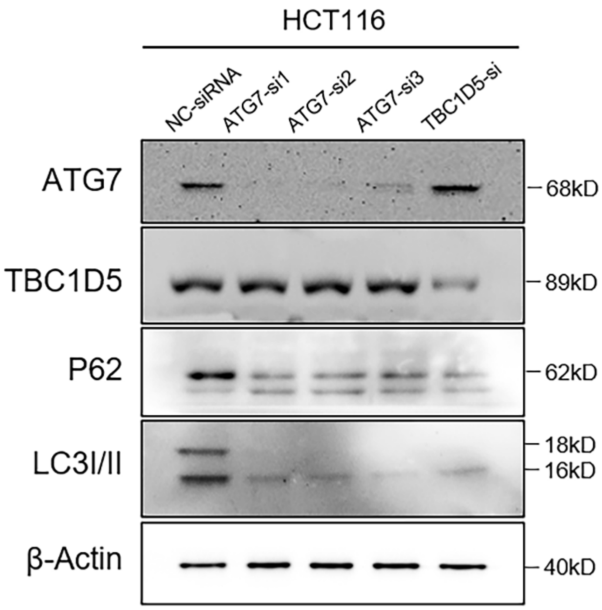

Supplement Figure 6 (A) HCT116 was transfected with NC-siRNA and TBK1-siRNA, TBK1, P62 and GAPDH were blotted. (B) HCT116 cells were transfected with NC-siRNA, TBK1-siRNA, vector plasmid and $TBK1^{WT}$ plasmid as indicated. WB analysis of P62, GLUT1 and GAPDH from whole-cell lysates.

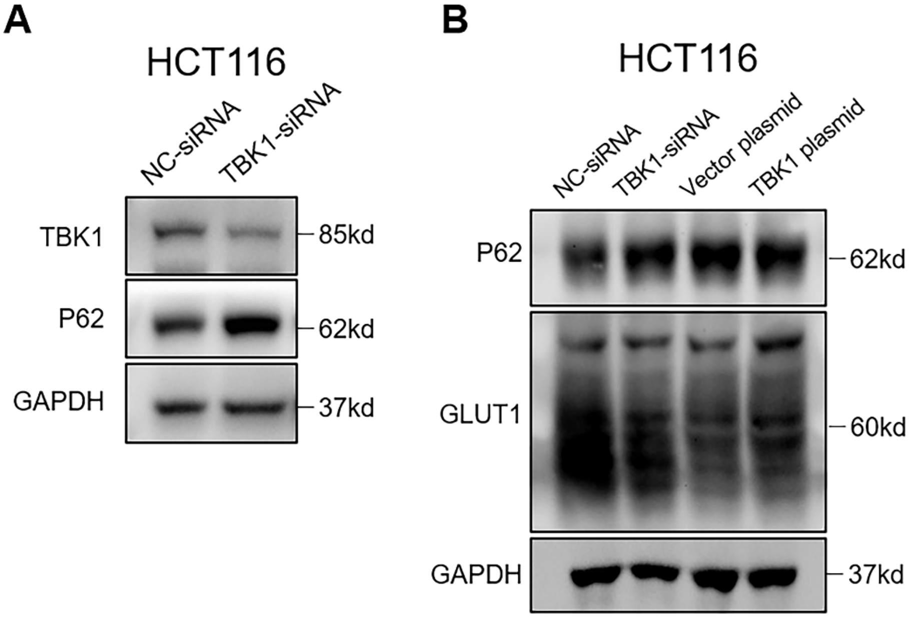

Supplement Figure 7 HCT116 and SW480 cells were treated with Amlexanox (100 μM) or poly (I:C) (2 μg/ml) for 24 h, and the lysates were blotted for P62, LC3I/II, GAPDH.

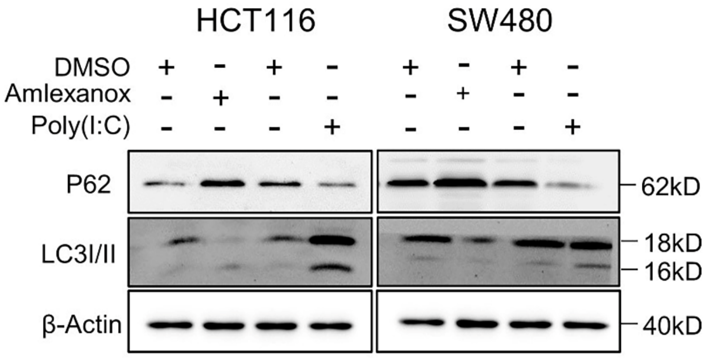

Supplement Table 1. siRNA sequences applied in the study

NC	Sense: 5’- UUCUCCGAACGUGUCACGUTT -3’	Sense: 5’- ACGUGACACGUUCGGAGAATT -3’
FAM NC	Sense: 5’- UUCUCCGAACGUGUCACGUTT -3’	Sense: 5’- ACGUGACACGUUCGGAGAATT -3’
TBK1-homo-247	Sense: 5’- CCAAGGAGCUACUGCAAAUTT -3’	Sense: 5’- AUUUGCAGUAGCUCCUUGGTT -3’
TBK1-homo-1310	Sense: 5’- GGGAACCUCUGAAUACCAUTT -3’	Sense: 5’- AUGGUAUUCAGAGGUUCCCTT -3’
TBK1-homo-1953	Sense: 5’-GACGCACUUUACAGAUGAATT-3’	Antisense: 5’-UUCAUCUGUAAAGUGCGUCTT-3’

Supplement Table 2. Primer sequences applied in the study

GLUT1	F: 5′-GGCCAAGAGTGTGCTAAAGAA-3′	R: 5′- ACAGCGTTGATGCCAGACAG-3′
mTOR	F: 5′-TCCGAGAGATGAGTCAAGAGG-3′	R: 5′-CACCTTCCACTCCTATGAGGC-3′
S6K1	F: 5′-AGAACTTCTGGCTCGAAAGGT-3′	R: 5′-CGACAGGTGTCTGACGTGTAA-3′
Raptor	F: 5′-AATGCTGCAATCGCCTCTTCT-3′	R:5′-CCAAAGGTAGGTTCCAGTCTG-3′
TBK1	F: 5′-ATGATTTAGACGGGGATGCTAG-3′	R: 5′-GAAATTTCACCTAACTCTGCCG-3′
18-S	F: 5′-GTAACCCGTTGAACCCCATT-3′	R: 5′-CCATCCAATCGGTAGTAGCG-3′
